# Establishment and maintenance of random monoallelic expression

**DOI:** 10.1242/dev.201741

**Published:** 2024-05-30

**Authors:** Eleni Kanata, Rachel Duffié, Edda G. Schulz

**Affiliations:** ^1^ Systems Epigenetics, Otto Warburg Laboratories, Max Planck Institute for Molecular Genetics, 14195 Berlin, Germany; ^2^ Department of Biochemistry and Molecular Biophysics, Mortimer B. Zuckerman Mind, Brain, and Behavior Institute, Columbia University, New York, NY 10027, USA

**Keywords:** X-chromosome inactivation, Epigenetic memory, Monoallelic expression, Olfactory receptors, Symmetry breaking

## Abstract

This Review elucidates the regulatory principles of random monoallelic expression by focusing on two well-studied examples: the X-chromosome inactivation regulator *Xist* and the olfactory receptor gene family. Although the choice of a single X chromosome or olfactory receptor occurs in different developmental contexts, common gene regulatory principles guide monoallelic expression in both systems. In both cases, an event breaks the symmetry between genetically and epigenetically identical copies of the gene, leading to the expression of one single random allele, stabilized through negative feedback control. Although many regulatory steps that govern the establishment and maintenance of monoallelic expression have been identified, key pieces of the puzzle are still missing. We provide an overview of the current knowledge and models for the monoallelic expression of *Xist* and olfactory receptors. We discuss their similarities and differences, and highlight open questions and approaches that could guide the study of other monoallelically expressed genes.

## Teaser

**Summary:** This Review provides an overview of the known regulatory mechanisms and current models for the establishment and maintenance of monoallelic expression, with *Xist* and olfactory receptors as case studies.

## Introduction

Transcription is regulated in a precise manner to allow a large diversity of cellular phenotypes, driven by distinct gene activity profiles. In part, expression patterns are directly controlled by cell type-specific trans-acting factors (e.g. transcription factors; TFs). These bind promoters and distal regulatory DNA elements, such as enhancers, to modulate promoter activity (Field and Adelman, 2020). The ability of these trans-acting factors to drive target gene expression is also controlled by regulatory events occurring *in cis* at the allele level. Here, chromatin remodeling complexes, post-translational modifications of histones, DNA modifications, as well as chromatin looping can promote or prevent gene activation (Bolt and Duboule, 2020; Ibrahim and Mundlos, 2020; Morgan and Shilatifard, 2020). This layer of control is often referred to as epigenetic regulation.

Stable monoallelic expression illustrates the power of epigenetic processes: sequence-identical copies of a gene respond differently to the same cellular environment, such that individual alleles can assume distinct transcriptional states. Genomic imprinting, where an allele is active or silent depending on the parent of origin, is a well-studied mode of monoallelic expression (Tucci et al., 2019). In other cases, the choice of the active allele is not deterministic, but occurs randomly in each cell, leading to a mixed population of cells in which different alleles are active (Gendrel et al., 2016). Well-studied examples of random monoallelic expression (RME) are olfactory receptors (ORs) (Chess et al., 1994), protocadherins in the nervous system (Tasic et al., 2002; Wang et al., 2002), and X-chromosomal genes in all somatic tissues of female mammals (Lyon, 1961). In the latter case, one out of two copies of a gene are active in each cell, whereas ORs and protocadherins are large gene families, where only one allele out of many is expressed in each cell (monogenic expression). Imprinted genes are differentially marked in the parental germ lines, where the two alleles are physically separated from each other. In contrast, the establishment of RME requires a symmetry-breaking event. More specifically, this is a process that allows two (or more) alleles of the same gene (or gene family) to acquire distinct molecular states within the same cell that ultimately lead to asymmetric transcription.

For genes that are expressed in a strict monoallelic manner (strict RME), which is the focus of this Review, each cell expresses exactly one copy of the gene or gene family stably, with all other alleles completely silent. This pattern is distinct from a broader class of facultative RME genes, such as the Ly49 family of receptors in natural killer (NK) cells, where a cell can stably express either none, one or both copies of a gene, suggesting independent regulation of each allele (Held et al., 1999). In contrast, strict RME genes use feedback mechanisms to ensure that exactly one allele is expressed per cell (Mutzel et al., 2019; Serizawa et al., 2003).

Most X-chromosomal genes are expressed monoallelically in female mammals as a result of X-chromosome inactivation (XCI), which compensates for double X-chromosome dosage (Loda et al., 2022). XCI is established during early embryogenesis, with the upregulation of the long non-coding RNA *Xist* from one randomly chosen X chromosome (Lyon, 1961). The two *Xist* alleles thus adopt opposing expression states that are stably maintained throughout mitotic divisions, keeping one of the two X chromosomes silenced (Brockdorff et al., 1991; Brown et al., 1991). Whereas each cell needs to choose between two copies of the *Xist* gene, the choice of ORs is an order of magnitude more challenging because each olfactory neuron expresses exactly one out of several thousand members of the OR gene family (Zhang and Firestein, 2002), potentially requiring a more elaborate system for symmetry breaking.

Random monoallelic expression can enrich the diversity within the cell population, with potential functional and evolutionary benefits (Chess, 2012). In humans, RME genes tend to exhibit higher mutation tolerance and evolvability, and offer phenotypic variation (Kravitz et al., 2023). In the case of ORs, their monoallelic and monogenic expression results in a multitude of specialized cells, permitting coverage of a large odorant space (Monahan and Lomvardas, 2015). The mosaicism created by random XCI is thought to offer an advantage for females, as they express a more diverse repertoire of proteins, and gain protection from potentially deleterious mutations on one of the two X chromosomes (Migeon, 2007).

In the next sections, we introduce the principles that are thought to govern the establishment of strict RME. We then summarize the current knowledge and proposed mechanisms for *Xist* and OR regulation, and draw parallels among them and other monoallelic paradigms.

## Regulatory principles of strict random monoallelic expression

**Fig. 1.**
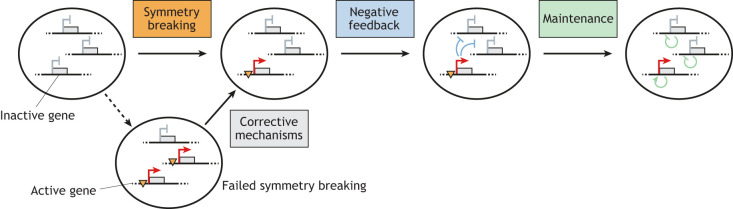
**Regulatory principles of strict random monoallelic expression (RME).** RME establishment can be divided into three conceptual phases. An initial symmetry-breaking event (orange triangle) allows transcriptional activation of one random allele. Corrective mechanisms are in place, in case initial symmetry breaking fails and more than one allele is activated. Activation of one random allele triggers negative feedback to prevent activation of other alleles. Once the correct allelic expression states have been established, they are maintained by self-reinforcing mechanisms that also allow inheritance through mitosis, if required.

To establish monoallelic expression, two or more initially similar copies of a gene must assume opposing expression states: one expressed, and the others silent (Fig. 1). For both ORs and *Xist*, a mechanistic concept has been suggested whereby an initial stochastic symmetry-breaking event results in activation of one allele (monoallelic expression), followed by negative feedback that prevents activation of the other allele(s) (Monkhorst et al., 2008; Tan et al., 2013). In this framework, the alleles are at first regulated independently of each other and only the negative feedback ensures coordination among them. Subsequent maintenance of the monoallelic state requires allele-specific mechanisms to stabilize active and inactive states in the same nucleus.

To allow robust symmetry breaking, the underlying molecular event(s) should be sufficiently stable to persist until the negative feedback is triggered. These events should also be sufficiently rare that they are unlikely to occur on the second allele prior to feedback activation. Symmetry breaking had therefore previously been suggested to occur over a much slower timescale than negative feedback (timescale separation) (Gartler and Riggs, 1983; Tan et al., 2013). However, more recent quantitative analyses indicate that they actually occur on rather similar timescales (McClintock et al., 2020; Shiura and Abe, 2019). As a consequence, symmetry breaking is heterogeneous, with a subset of cells failing to break the symmetry initially, resulting in co-expression of multiple gene copies. Additional ‘corrective’ mechanisms therefore exist to ensure that all cells eventually reach the monoallelic state (Fig. 1). Allelic independence together with corrective mechanisms might help for robust decision making. Even in cases in which one allele is non-functional, all cells will reach a monoallelic state.

## *Xist* and XCI

**Fig. 2.**
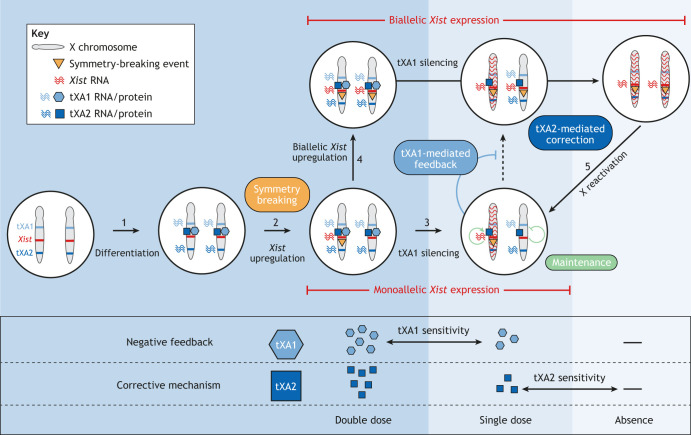
**The establishment of monoallelic *Xist* expression.** During the onset of random X-chromosome inactivation in mice, cells can reach the monoallelic *Xist* expression state via two different routes. (1) Upon differentiation, *trans*-acting *Xist* activators (tXA1 and tXA2), are expressed. (2) In most cells, a symmetry-breaking event (orange triangles) triggers monoallelic expression of *Xist*, the long non-coding RNA that covers the X chromosome and silences it. (3) Monoallelic *Xist* upregulation is stabilized through negative feedback, which is mediated by the silencing of tXA1. Additional mechanisms maintain the monoallelic silencing of one chromosome (green). (4) In a subset of cells, *Xist* upregulation from the second allele occurs before tXA silencing, which results in biallelic *Xist* expression. (5) Here, a corrective mechanism is in place, triggered by biallelic silencing of tXA2 by *Xist*, causing *Xist* downregulation and reactivation of one X chromosome. For negative feedback, the *Xist* locus must be sensitive to changes in tXA1 from double to single dose, while the corrective mechanism is triggered by the absence of tXA2 factors. tXA1 and tXA2 might be the same or different factors. Background colors indicate the state-associated X dosage.

XCI leads to chromosome-wide gene silencing on one random X chromosome. This process is initiated specifically in females during early embryonic development by the upregulation of *Xist* from a single X chromosome (Loda et al., 2022). The *Xist* RNA spreads *in cis* across the chromosome and recruits silencing factors (Loda et al., 2022). Female-specificity of *Xist* expression is, at least in part, promoted by increased levels of X-linked *Xist* activators in females compared with males (Monkhorst et al., 2008) (Fig. 2). Once symmetry breaking has occurred through stochastic *Xist* upregulation, negative feedback is triggered by *Xist*-mediated silencing of X-chromosomal genes: reduced levels of X-linked *Xist* activators potentially prevent *Xist* upregulation from the second allele (Mutzel and Schulz, 2020). Once established, the silent and active states of the two *Xist* alleles are stably maintained throughout all further cell divisions. Given that *Xist* regulation has been predominantly investigated in mice, statements herein generally refer to the murine system unless otherwise specified. An overview of known *Xist* regulators is provided in Box 1.

**Box 1. *Xist* regulation**

The *Xist* gene lies within a regulatory locus termed the X-inactivation center, which contains several regulatory long non-coding RNAs that either activate (*Jpx*, *Ftx*, *Xert*) or repress (*Tsix*, *Linx*) *Xist*. *Tsix*, *Xist's* antisense transcript, represses *Xist in cis*, through transcriptional interference possibly mediated by polymerase collision and by deposition of repressive chromatin modifications (Mutzel et al., 2019; Navarro et al., 2005; Ohhata et al., 2008, 2021; Sado et al., 2005). Several *Xist* regulators bind the promoter-proximal region, such as the *Xist*-activating transcription factor YY1 (Makhlouf et al., 2014), the replication-timing regulator RIF1 (Enervald et al., 2021), the *Xist*-repressor REX1 (ZFP42) (Gontan et al., 2012) and CTCF (Pugacheva et al., 2005). REX1 is thought to be degraded in an X-dosage-dependent manner by the X-linked E3 ubiquitin ligase RNF12 (RLIM), which may contribute to female-specific *Xist* expression. Because *Xist* is upregulated when cells leave the naïve pluripotent state, naïve pluripotency factors, such as REX1, NANOG and PRDM14, repress *Xist* (Donohoe et al., 2009; Gontan et al., 2012; Navarro et al., 2008; Payer et al., 2013), and epiblast-associated factors, such as OTX2 and SMAD2/3, might activate *Xist* by binding to its enhancers (Gjaltema et al., 2022). TAD, topologically associated domain.
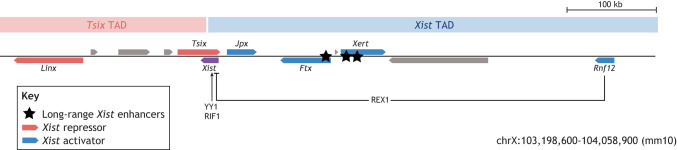

### *Xist*, the master regulator of XCI

**Fig. 3.**
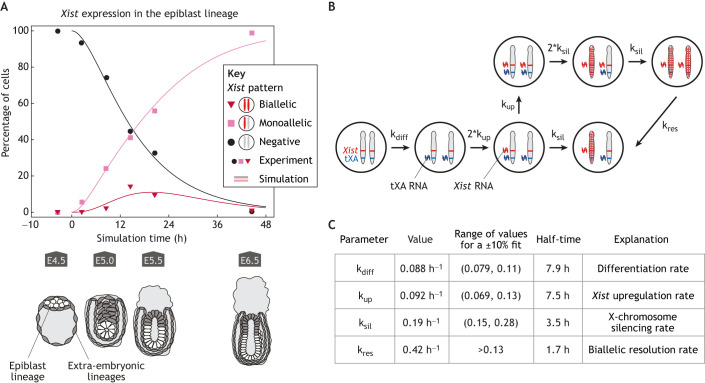
**The dynamics of random *Xist* upregulation *in vivo*.** (A) Dynamics of *Xist* upregulation in the epiblast lineage during mouse development (Shiura and Abe, 2019). Cells are categorized according to their *Xist* expression pattern measured by RNA fluorescence *in situ* hybridization (monoallelic, biallelic or negative, as indicated), and the corresponding developmental stages are indicated below the plot (cells of the epiblast lineage, where random X-chromosome inactivation occurs, are labeled in white). Symbols indicate the experimental data and lines a simulation, using the model and parameters shown in B and C. (B) Schematic representation of the mathematical model used to estimate timescales of *Xist* upregulation (symmetry breaking) and negative feedback (silencing) from the data shown in A. A system of ordinary differential equations with one variable describing each of the indicated cell states (see Fig. 2 for details) was fitted to the data to estimate the rates with which cells transition between states, which are given by four different parameters shown in C. As an additional parameter, the starting point of the simulation was optimized (0 h=E4.6). (C) Fitted parameter values of the model in B that best explain the experimental data shown in A. An initial differentiation step had to be assumed to reproduce the data that renders cells competent for *Xist* upregulation (rate given by k_diff_). From the competent state, each allele can upregulate *Xist* with probability k_up_, resulting in X-chromosomal silencing with rate k_sil_. In the case of biallelic silencing, a corrective mechanism is assumed to be triggered, leading to the resolution of the biallelic state with the rate k_res_. For each parameter, the fitted value and an interval, where the goodness of fit decreases by <10% (range) are given. Moreover, the associated half-time, calculated as ln(2)/k, is indicated, which represents the time it takes until the event has occurred in 50% of cells/alleles. *Xist* upregulation (symmetry breaking) and X-linked activator silencing (feedback) occur on similar timescales, leading to a mix of monoallelic and biallelic cells.

Mice undergo two waves of XCI: imprinted XCI, which occurs in rodents, but probably not in other placental mammals, and random XCI (rXCI). During imprinted XCI, all cells inactivate the paternal X chromosome because of an H3K27me3 imprint on the maternal *Xist* allele, which keeps *Xist* repressed (Inoue et al., 2017). Imprinted XCI is initiated at the 4-cell stage and is maintained in the extra-embryonic lineages. In the epiblast lineage, which gives rise to the embryo, *Xist* is downregulated by embryonic day (E) 4.5 (Shiura and Abe, 2019) and reactivation of X-chromosomal genes is complete by E5.5 (Cheng et al., 2019). The second wave of XCI begins with *Xist* upregulation around Ε4.75, now randomly, with virtually all cells reaching a monoallelic state within 2 days (Fig. 3) (Shiura and Abe, 2019). The onset of rXCI can be modeled in differentiating embryonic stem cells (ESCs). To ensure monoallelic expression, stochastic *Xist* upregulation (symmetry breaking) leading to XCI on one allele is thought to trigger negative feedback by silencing X-linked *Xist* activators (Monkhorst et al., 2008). *Xist* remains expressed in all somatic cells, where it supports the maintenance of X-linked gene silencing. Its loss after XCI establishment only partially reactivates the X chromosome because other mechanisms, such as promoter DNA methylation, have been established and ensure silencing (Richart et al., 2022; Yang et al., 2016; Yu et al., 2021).

In Fig. 3, we use a simple mathematical model to extract the timescales of symmetry breaking (*Xist* upregulation) and negative feedback (activator silencing) by fitting the model to a published *in vivo* time-course dataset (Shiura and Abe, 2019). The symmetry-breaking event occurs once in 10 h on each allele (k_up_), and the feedback is triggered within ∼4 h once *Xist* is upregulated (k_sil_). With the feedback approximately twice as fast as the symmetry-breaking event, the timescale separation is not pronounced. As a consequence, *Xist* upregulation occurs on the second allele before feedback is triggered in a substantial fraction of cells (∼20%). This results in biallelic *Xist* expression (failed symmetry breaking), as also observed in other studies *in vivo* and *in vitro* (Guyochin et al., 2014; Mutzel et al., 2019; Pacini et al., 2021; Shiura and Abe, 2019; Sousa et al., 2018). Given that biallelic expression is only transient, a corrective mechanism seems to be in place, potentially mediated by complete *Xist*-activator silencing (see ‘Negative feedback in Xist regulation’ section below).

### Symmetry breaking at the *Xist* locus

As mentioned earlier, the XCI symmetry-breaking event must occur infrequently, but be stable for several hours. Individual regulatory events, such as TF binding or enhancer–promoter (E–P) contacts, seem to be too short-lived to allow symmetry breaking on these timescales (Box 2). However, a series or a combination of events might directly or indirectly activate or silence *Xist*, together making symmetry breaking sufficiently rare and stable.

**Box 2. Timescales of regulatory processes of gene regulation**

Most biochemical reactions involved in gene regulation occur on a timescale of seconds to minutes (Meeussen and Lenstra, 2024). On their own, individual reactions cannot account for a rare and stable symmetry-breaking event on a timescale of hours. Abundant transcription factors (TFs) stay bound to their target site for 10-100 s (Mazzocca et al., 2021). CTCF-cohesin loops, which often mediate enhancer-promoter (E–P) interactions, have a lifespan of 10–30 min (Gabriele et al., 2022; Mach et al., 2022). TF binding or E–P contacts can trigger a transcriptional burst, which can last from minutes to an hour (Suter et al., 2011), and/or promote a change in chromatin state. Histone modifications can be stable for hours to days (Lammers et al., 2020). A subset of histone modifications, in particular the repressive marks H3K9me3, H3K27me3 and H2AK119ub1, can be propagated *in cis*, based on read-write mechanisms, and thus stabilize the chromatin state for days to weeks (Hathaway et al., 2012; Holoch et al., 2021; Moussa et al., 2019).

An initial trigger for symmetry breaking could be stochastic TF binding, although this is usually short-lived (Box 2). YY1, RIF1 and CTCF bind at the *Xist* locus asymmetrically on the two alleles (Enervald et al., 2021; Makhlouf et al., 2014; Sun et al., 2013). For YY1 and CTCF, however, this pattern has been attributed to differential DNA methylation of the promoter-proximal region of *Xist* (Makhlouf et al., 2014), suggesting a preceding symmetry-breaking event. Another regulatory layer that might contribute to symmetry breaking is *Xist's* repressive antisense transcript, *Tsix* (Box 1). Although it is often co-expressed with *Xist* at the onset of rXCI (E5.0) (Shiura and Abe, 2019), fluctuations in *Tsix* transcription could initiate monoallelic *Xist* upregulation because *Tsix* is a strong repressor of *Xist in cis* (Aeby et al., 2020). Heterozygous deletions of *Tsix* lead to a fast, seemingly deterministic choice of the inactive X, with *Xist* being upregulated from the *Tsix*-mutant allele (Lee and Lu, 1999), and a homozygous mutation results in more frequent and sustained biallelic *Xist* expression (Lee, 2005). Whether *Tsix* is required for symmetry breaking in the wild-type context remains to be shown. Notably, *Tsix* expression might not be conserved in other mammals because, to our knowledge, it has not been detected in non-rodent embryos, pointing towards additional symmetry-breaking mechanisms.

Stochastic contacts with long-range enhancers could also be involved in symmetry breaking. This seems to be the case in the slow stochastic upregulation of *Bcl11b*, where contacts between the promoter and an enhancer ∼85 kb away control upregulation independently on each allele (Ng et al., 2018). A systematic analysis of facultative RME genes through allele-specific ATAC-seq (assay for transposase-accessible chromatin with sequencing) has shown that enhancers are usually accessible on both alleles, whereas promoters exhibit allelic accessibility patterns (Xu et al., 2017). The gene family encoding NK receptors follows this pattern: whereas their enhancers are active on all alleles, promoters are only active on a subset, but the activation probability is reduced upon enhancer deletion (Kissiov et al., 2022). These studies suggest that symmetry breaking does not occur on the level of enhancer activation. Instead, stochastic E–P contacts likely drive promoter activation on one allele. At the *Xist* locus, distal enhancers of *Xist* are also active on both the *Xist*-expressing and the *Xist*-silent allele (Gjaltema et al., 2022). CRISPR interference (CRISPRi)-mediated repression of individual enhancer elements reduces the fraction of cells that upregulate *Xist* (in addition to the expression level per cell), suggesting that E–P contacts modulate the probability of *Xist* upregulation (Gjaltema et al., 2022). Interestingly, long E–P distances (above 100 kb) increase cell-to-cell variability of gene expression (Zuin et al., 2022). The distance between *Xist* and some of its enhancers (∼140 kb away) might favor stochastic interactions that would promote symmetry breaking.

In summary, the data point towards a model whereby *Xist* transcription is part of the initial symmetry breaking, potentially triggered by stochastic E–P contacts or fluctuations in *Tsix* transcription. Additional layers of regulation nevertheless might be involved, such as nuclear organization. Specifically, the spatial proximity of the two X chromosomes, termed X-chromosome pairing, had been suggested as a symmetry-breaking event in the past (Bacher et al., 2006; Xu et al., 2006), but preventing X-pairing by tethering one allele to the nuclear periphery does not affect choice (Barakat et al., 2014; Pollex and Heard, 2019). The quest for the symmetry-breaking mechanism has, thus far, focused on perturbing and observing asymmetric events between the two alleles. Yet, discerning whether the asymmetries are the cause or consequence of monoallelic expression is challenging, and an altered XCI phenotype upon perturbation of a regulatory mechanism does not conclusively implicate its involvement in endogenous symmetry breaking. In the future, live-cell imaging of *Xist* upregulation together with potential upstream symmetry-breaking events, such as DNA looping or transcription of *cis*-acting *Xist* regulators such as *Tsix*, will be required to test which events actually precede monoallelic *Xist* upregulation.

### Negative feedback in *Xist* regulation

Mary Lyon, who discovered X inactivation in 1961, proposed a mechanism for negative feedback: the silencing of an X-linked XCI activator after initiation of XCI on one X might prevent silencing of the other X chromosome (Lyon, 1971). Such an X-linked XCI activator is thought to be a *trans*-acting factor that can activate *Xist* in a dosage-sensitive fashion and is silenced by the *Xist* RNA during XCI (Monkhorst et al., 2008). This activator must be capable of initiating *Xist* upregulation only when present at a double dose (XX). *Xist*-dependent silencing of the activator would reduce its dose from double to single, which would be too low to initiate Xist upregulation on the second allele (Fig. 2). This is only true for normal diploid cells, however. Female tetraploid cells appear to trigger the feedback only when two X chromosomes out of four have been inactivated, resulting in stable inactivation of two X chromosomes (Monkhorst et al., 2008; Webb et al., 1992). The underlying mechanisms are unknown, but might involve dosage-sensitive autosomal *Xist* regulators or a dilution of X-linked factors due to increased nuclear volume (Gartler and Riggs, 1983; Mutzel et al., 2019).

The only known X-linked *Xist* activator that is subject to XCI is the E3 ubiquitin ligase RNF12 (RLIM), which ubiquitinates the *Xist* repressor REX1 (ZFP42) and targets it for degradation (Gontan et al., 2012, 2018; Jonkers et al., 2009). *Rnf12* is among the most rapidly silenced X-linked genes (∼6 h silencing half-time), making it a suitable candidate for triggering feedback (Barros de Andrade et al., 2019). In mouse ESCs *in vitro*, a homozygous *Rnf12* deletion strongly impairs XCI (Barakat et al., 2011, 2014; Gontan et al., 2018; Wang et al., 2017). Whether *Xist* responds to RNF12 dosage alteration (which would be predicted for a feedback regulator) is less clear, because the deletion of one *Rnf12* copy in female ESCs does not abrogate, only delays, *Xist* upregulation (Jonkers et al., 2009). *In vivo*, however, RNF12 seems to be dispensable for the onset of rXCI (Gontan et al., 2018; Shin et al., 2014). This observation suggests either redundant mechanisms, which remain to be discovered, that ensure XCI feedback, or a diminished role of the RNF12/REX1 pathway. The latter could be attributed to the transcriptional downregulation of REX1 at that developmental stage (Wang et al., 2023).

Although additional X-linked factors might be involved in triggering negative feedback, there is some evidence that the RNF12/REX1 axis mediates a corrective mechanism in cells when initial symmetry breaking fails and *Xist* is upregulated from both chromosomes (Mutzel et al., 2019). Here, the complete absence of RNF12 leads to REX1 stabilization, which in turn represses *Xist*, resulting in its downregulation (Fig. 2). This idea is supported by several observations: (1) *Rex1* homozygous mutants (absence of RNF12-mediated feedback) show increased levels of biallelic *Xist* expression (Gontan et al., 2018); (2) *Rnf12*-heterozygous mutants always inactivate the mutated X (because silencing of the wild-type allele leads to complete absence of RNF12 protein and *Xist* downregulation) (Jonkers et al., 2009); (3) a heterozygous deletion of *Xist* and *Rnf12* on the same allele prevents *Xist* upregulation (likely because XCI would lead to a complete loss of RNF12 protein), but cells can initiate XCI when the deletions are introduced on two different alleles (Barakat et al., 2014). Reversal of *Xist* upregulation upon complete *Rnf12* silencing could even explain the delay in XCI onset observed in the heterozygous mutant cells mentioned above (Jonkers et al., 2009). Here, the inactivation of the wild-type allele cannot be sustained owing to the resulting absence of RNF12 protein (see point 2 above) and cells initially inactivating that allele might need more attempts of stochastic *Xist* upregulation until they eventually inactivate the mutant allele.

A corrective mechanism might only be important in a minority of cells in mice (∼20% biallelic *Xist*), but might play a more prominent role in other mammals, in which transient biallelic *Xist* expression is more frequent (∼50% in rabbits, 80-90% in primates) (Okamoto et al., 2011, 2021; Petropoulos et al., 2016). In primates, the biallelic state is present over an extended period (>3 days) during which *Xist* is not yet competent to silence X-linked genes (Okamoto et al., 2021; Petropoulos et al., 2016). The transition to monoallelic expression occurs when *Xist* becomes fully competent to silence the X chromosome (Okamoto et al., 2021), possibly relying on the RNF12-mediated corrective mechanism. What happens once the corrective mechanism is triggered? Co-staining of *Xist* RNA and *Xist*-dependent accumulation of H3K27me3 on the X chromosome in differentiating mouse ESCs and in primate embryos has revealed that cells with biallelic H3K27me3 accumulation can exhibit monoallelic *Xist* expression, but are never *Xist* negative (Mutzel et al., 2019; Okamoto et al., 2021). This suggests that cells transition directly from the biallelic to a monoallelic state. Thus, symmetry breaking does not only occur through stochastic *Xist* upregulation, but also through random downregulation.

Finally, additional mechanisms exist to ensure that all cells in a female embryo have undergone XCI. Because several X-linked genes (e.g. *Dusp9*, *Klhl13*) counteract cellular differentiation when present in a double dose, successful XCI will trigger further differentiation (Genolet et al., 2021; Schulz et al., 2014). If cells are unable to reach the monoallelic state over extended periods, this might lead to apoptosis or replicative senescence. This has been observed for cells that have more inactive X chromosomes than required after 7 days, but not after 2 days of mouse ESC differentiation, when transient biallelic *Xist* expression occurs normally (Monkhorst et al., 2008; Mutzel et al., 2019). Cell death or senescence in cells with too many inactive X chromosomes might thus be a fallback mechanism in case biallelic expression is not fully resolved, and may rely on the same mechanisms that eliminate aneuploid cells (Singla et al., 2020).

### Maintenance of monoallelic *Xist* expression

As the inactive X is established during early embryogenesis and inherited through all further cell divisions during the lifetime of the organism, the alternative *Xist* expression states must be faithfully inherited through mitosis (memory). The mechanisms of memory must therefore act *in cis*, to be allele-restricted and clonally heritable.

One proposed mechanism is *Xist*-mediated silencing of an *Xist* repressor, which would keep *Xist* silent on one allele, but allow expression from the other (Mutzel et al., 2019). An obvious candidate is *Tsix*, because it is a well-characterized *cis*-repressor and silenced by *Xist*. As *Tsix* is essentially undetectable 2.5 days after rXCI onset (Panning et al., 1997; Sheardown et al., 1997; Shiura and Abe, 2019), and is silent in many somatic tissues (Li et al., 2017), additional mechanisms are required for long-term memory. *Tsix* transcription initiates a cascade of changes on the *Xist*-negative allele (active X), which leads to heritable memory, including H3K9me3 and DNA methylation (Gjaltema et al., 2022; Navarro et al., 2005; Norris et al., 1994; Ohhata et al., 2021; Sado et al., 2005). Interestingly, both DNA methylation and H3K9me3 are also hallmarks of canonical imprinted genes (Monk et al., 2019). In particular, DNA methylation is a central mechanism of epigenetic memory because it can be faithfully inherited through cell divisions (Li et al., 1992). However, loss of DNA methylation leads to derepression of *Xist* at the active X only in a minority of cells at E9.5 (Beard et al., 1995; Sado et al., 2004).

The repressive histone mark H3K9me3 can mediate epigenetic memory because it can be stably maintained through cell divisions in the absence of the initial signal (Hathaway et al., 2012) through self-reinforcing read-write mechanisms (Grewal, 2023). Whether H3K9me3 can maintain memory in the absence of DNA methylation, or whether additional mechanisms are involved in maintenance, remains to be determined. Interestingly, many facultative RME genes, including NK receptors, do not show differential marking for repressive chromatin modifications (e.g. DNA methylation, H3K9me3, H3K27me3) but only for active marks (e.g. H3K4m3, H3K27ac) at the promoter and the gene body of the active allele(s) (Balasooriya and Spector, 2022; Kissiov et al., 2022). For these marks, it is less clear whether and how they can be inherited through cell division.

Asynchronous replication timing, frequently associated with monoallelic expression, might also contribute to the maintenance of two transcriptional states (Bergman et al., 2021). The *Xist* locus remains early-replicating on both chromosomes after XCI onset (Poonperm et al., 2023), implying that if asynchronous replication serves a role for maintenance, the signal would come from another region on the X chromosome that is asynchronously replicated. Another maintenance mechanism of RME genes is localization in different nuclear compartments (Takizawa et al., 2008), but the functional importance of nuclear organization for long-term maintenance of *Xist* expression remains to be investigated.

Symmetry breaking, feedback and maintenance of the chosen allele govern both *Xist* locus and OR choice. The following section summarizes the current state of knowledge about monoallelic OR expression, to then compare the two systems.

## The olfactory system

**Fig. 4.**
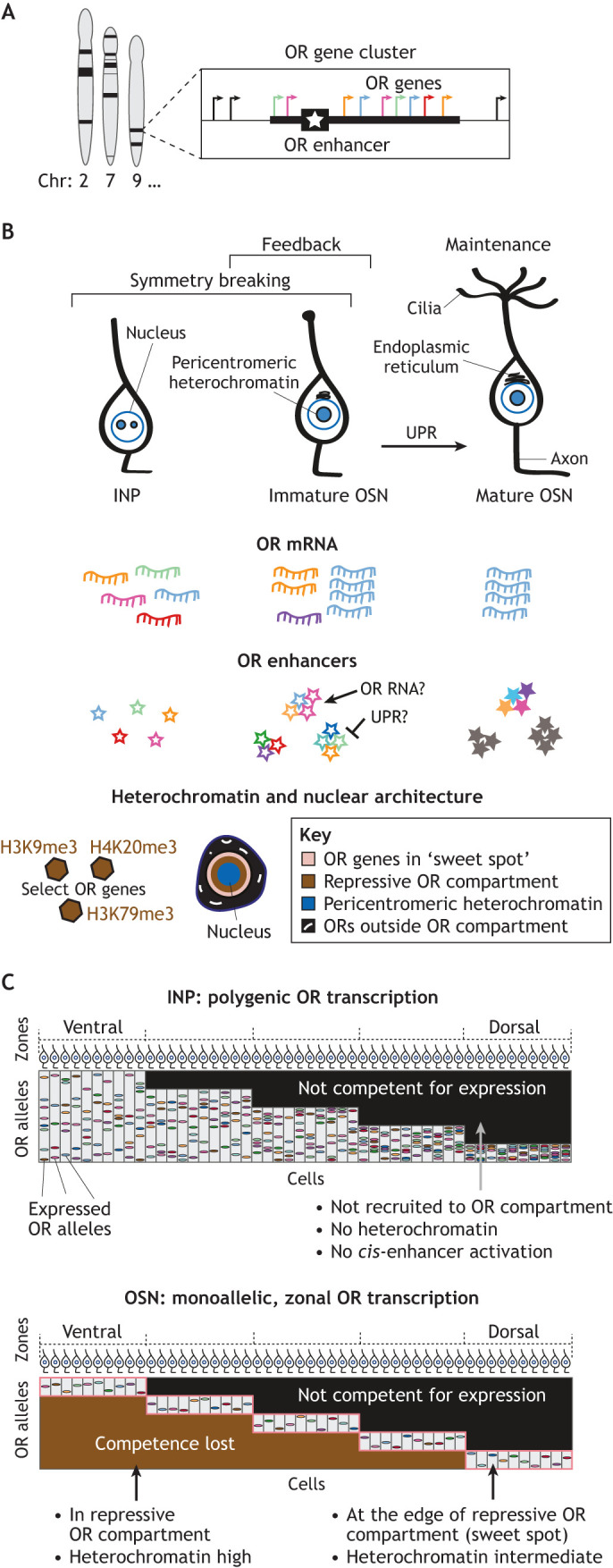
**Olfactory receptor regulation.** (A) Olfactory receptor (OR) genes (shown in various colors) are located in multiple clusters spread across chromosomes with approximately one OR enhancer (star) per cluster. (B) Model of OR choice in the context of neuronal differentiation. Symmetry breaking occurs at the immediate neuronal progenitor (INP) and immature olfactory sensory neuron (OSN) stage, with zone-specific (see C) polygenic OR transcription in INP cells coupled with heterochromatin formation and nuclear rearrangement of OR genes. Previously thought to apply to all ORs in the cell, recent work highlights that only select ORs are coated in heterochromatin and recruited to OR compartments based on zonal expression patterns. The number of transcribed ORs becomes progressively restricted as cells transition to the OSN stage, initiated by high transcription of an OR upon interaction with an enhancer hub (unfilled stars). An active enhancer hub (filled colored stars) emerges from several enhancer hubs per cell, potentially as OR RNA prevents activation of other OR alleles. Negative feedback is mediated by UPR in immature OSNs and prevents activation of other OR alleles. Only the expressed OR alleles are at the edge of the OR nuclear compartment in the ‘sweet spot’ for expression, whereas repressed alleles are buried closer to pericentromeric heterochromatin. (C) Progressive zonal restriction of OR competence upon transition from the INP to the mature OSN stage. Columns represent cells and rows OR alleles, with colored stripes indicating expressed alleles. ORs in light gray segments in the OSN stage have intermediate levels of heterochromatin and intermediate OR-OR *trans* contacts, and would be positioned in the rose-colored ‘sweet spot’ in B.

The olfactory system has the daunting task of detecting innumerable volatile odorants in the environment and translating those chemicals into meaningful information (Bushdid et al., 2014). To ensure a wide receptive field to the myriad of odorants in the environment, mammalian genomes encode hundreds to thousands of OR genes (Barnes et al., 2020; Buck and Axel, 1991; Niimura et al., 2014) (Fig. 4A). This gene family is exquisitely regulated in the cells where they are expressed: the olfactory sensory neurons (OSNs). Each mature OSN expresses one OR monogenically (one gene of the OR family) and monoallelically (one allele of the chosen OR gene) to allow for precise encoding of olfactory information (explained in greater detail in Box 3) (Chess et al., 1994; Serizawa et al., 2000).

**Box 3. Olfactory receptor choice and axonal wiring**

Perhaps the most important biological reason for monoallelic olfactory receptor (OR) expression is to ensure that the olfactory sensory neuron (OSN) wires to the correct glomerulus in the olfactory bulb. The chosen OR defines the receptive field of the neuron and leads the OSN to depolarize in a predictable pattern upon detection of odorants (Malnic et al., 1999; Nara et al., 2011; Xu et al., 2020). Across the tissue, the cells choosing the same OR project their axons to the same glomeruli in the olfactory bulb, leading to a spatial representation of odors in the bulb (Ressler et al., 1994; Sakano, 2020; Wang et al., 2022). Patterns of OSN neuronal activity are thus translated into a map of odorants in the brain (Mombaerts et al., 1996; Vassar et al., 1994).

Recent work has uncovered a molecular explanation for how singular choice instructs precise axonal wiring. Each OR activates the unfolded protein response to varying degrees, which can be measured by ATF5 translation. Neurons that choose an ATF5^high^ OR have a different suite of axonal guidance molecules than OSNs that choose an ATF5^low^ OR. DDIT3 has been identified as a key UPR sensitive transcription factor that orchestrates the axonal wiring ‘barcodes’ of OSNs choosing a specific OR (Shayya et al., 2022). OR-specific axon guidance transcriptional profiles have also been independently identified in an elegant study using spatial transcriptomics (Wang et al., 2022). Singular OR choice thus governs the levels of axon guidance molecules in the cell to ensure a defined map of odors in the brain.

OSN stem cells, capable of regenerating throughout life, differentiate from immediate neuronal progenitors (INPs), to immature OSNs to mature OSNs (Fig. 4B) (Fletcher et al., 2017; Hanchate et al., 2015; Huard et al., 1998; McClintock et al., 2020). During the INP stage, five to ten ORs are expressed per cell, termed polygenic OR transcription (Hanchate et al., 2015; Saraiva et al., 2015; Tan et al., 2015). As OSNs differentiate, layers of gene regulatory processes work in harmony to ensure that just one OR allele is expressed in each mature OSN (Fig. 4B). These regulatory programs are modulated by neuronal differentiation, and in turn singular OR choice allows a neuron to reach its mature state (Dalton et al., 2013; Lyons et al., 2013).

### Layers of gene regulation governing OR expression

OR genes have peculiar genomic organization and sequence composition (Brovkina et al., 2023). The mouse genome encodes approximately 1400 OR genes, which are arranged across 18 chromosomes in large genomic clusters (Fig. 4A) (Barnes et al., 2020; Ben-Arie et al., 1994; Godfrey et al., 2004). OR promoter elements are AT-rich, lack CpG islands and are enriched for MEF2A, LHX2 and EBF motifs relative to random sequences of the same AT/GC composition (Clowney et al., 2011; Michaloski et al., 2006; Plessy et al., 2012). Most, if not all, OR clusters harbor an OR enhancer that comes together with other OR enhancers *in cis* and *in trans* to form an OR enhancer hub (Box 4).

**Box 4. Olfactory receptor enhancer network**

Most – or all – olfactory receptor (OR) gene clusters harbor an OR enhancer (Monahan et al., 2017; Wu et al., 2024). Enhancers are identified as a class by their enrichment for DNA accessibility, H3K4me1 and flanking H3K27ac histone modifications (Markenscoff-Papadimitriou et al., 2014). A ‘composite motif’ for LHX2 and EBF transcription factors in these enhancers is essential for OR activation (Monahan et al., 2017). *Cis* interactions between enhancers and OR promoters begin during the immediate neuronal progenitor stage (Pourmorady et al., 2024; Wu et al., 2024), and likely predispose an OR for choice. Deleting these *cis* elements leads to reduced transcription from proximal ORs, highlighting the important role of *cis* enhancers in OR activation (Fuss et al., 2007; Khan et al., 2011; Nishizumi et al., 2007; Serizawa et al., 2003).

Nuclear reorganization during differentiation facilitates *trans* interactions among OR enhancers (Lomvardas et al., 2006; Markenscoff-Papadimitriou et al., 2014; Monahan et al., 2019; Tan et al., 2019). Lim domain binding protein 1 (LDB1) binds LHX2 and stabilizes these long-range chromosomal interactions, allowing an enhancer hub to form over a transcriptionally active OR (Monahan et al., 2019). Three to nine OR enhancers from multiple chromosomes form a hub, but any combination of the 63-90 OR enhancer elements can act redundantly (Bashkirova et al., 2023; Pourmorady et al., 2024; Wu et al., 2024). DNA fluorescence *in situ* hybridization and single-cell HiC have revealed that three to eight enhancer hubs form per cell during the immediate neuronal progenitor and immature olfactory sensory neuron stages (Pourmorady et al., 2024; Tan et al., 2019), indicating that unknown mechanisms result in an a single active hub. The OR enhancer network is crucial for symmetry breaking, is likely a target of feedback, and may be important for the maintenance of OR choice.

Beginning in the INP stage, ORs are coated with repressive chromatin marks such as H3K9me3, H4K20me3 and H3K79me3 (Bashkirova et al., 2023; Magklara et al., 2011). It is currently unknown what seeds heterochromatin on OR clusters and which methyltransferases establish these patterns. Heterochromatin ‘levels the playing field’ so that all ORs have the propensity to be chosen (Lyons et al., 2014), and locks away OR genes that have not been chosen (Bashkirova et al., 2023). Heterochromatin modifications also govern nuclear architecture in OSNs, which has a profound effect on OR choice (Clowney et al., 2012; Le Gros et al., 2016).

Lamin B receptor and lamin A, which tether heterochromatin to the periphery of the nucleus, are downregulated during neuronal differentiation (Clowney et al., 2012; Yoon et al., 2015), causing the majority of heterochromatin to collapse into dense foci in the center of the nucleus (Armelin-Correa et al., 2014; Clowney et al., 2012; Le Gros et al., 2016). Heterochromatin-coated OR loci are recruited to these repressive nuclear compartments (Bashkirova et al., 2023; Markenscoff-Papadimitriou et al., 2014; Tan et al., 2019), locking away non-selected ORs to prevent their spurious expression (Bashkirova et al., 2023; Clowney et al., 2012). Surprisingly, recruitment to repressive OR compartments is also required for OR activation to bring OR loci in proximity to OR enhancers *in trans* (Markenscoff-Papadimitriou et al., 2014; Monahan et al., 2019; Tan et al., 2019). The OR enhancer network is another unique regulatory layer that guides monoallelic OR choice (Box 4).

### OR symmetry breaking

How does an OSN successfully express one chemoreceptor allele amidst thousands of similar possibilities? The process of symmetry breaking in OSNs can be distilled to three major levels: (1) chemoreceptor type; (2) zonal OR choice; and (3) random OR choice. Chemoreceptor types include ORs, which are further subdivided into type I ORs (Glusman et al., 2001) and type II ORs (Buck and Axel, 1991), trace amine-associated receptors (TAARs) (Liberles and Buck, 2006), guanylate cyclase D receptors (Bloom et al., 2020; Greer et al., 2016), and transient receptor potential 2 (Trpc2) receptors (Omura and Mombaerts, 2014). The molecular programs that determine which chemoreceptor subtype will be expressed in a cell merit further investigation, but must include developmentally timed and anatomically restricted cues (Bloom et al., 2020; Pacifico et al., 2012). The TF BCL11B promotes type II OR expression over type I OR expression (Enomoto et al., 2019), and other TFs likely modulate differentiation towards other chemoreceptor types. Although this Review focuses on type II OR regulation, it is interesting to note that clustered genomic organization, *cis*-linked enhancers and nuclear positioning all regulate type I ORs and TAARs, suggesting common regulatory principles for monoallelic receptor expression in OSNs (Brovkina et al., 2023; Fei et al., 2021; Iwata et al., 2017; Shah et al., 2021; Yoon et al., 2015).

#### Zonal OR choice

Zonal OR expression is a deterministic process that narrows down which ORs are competent to be expressed in an anatomical zone (Ressler et al., 1993; Strotmann et al., 1992; Vassar et al., 1993). A given OR has a distinct zonal identity: it can be expressed in one of five to nine anatomical regions organized along the dorsoventral axis of the olfactory epithelium in mice (Fig. 4C) (Bashkirova et al., 2023; Tan and Xie, 2018). Zonality is at least partly explained by repressive *cis* regulatory elements that are independent of the OR coding sequence (Qasba and Reed, 1998; Wang et al., 1998). *Cis* elements may also recruit TFs: the NFI TFs are highly expressed in ventral olfactory zones and are required for ventral OR identity (Bashkirova et al., 2023).

OSNs from different zones exhibit extensively different heterochromatin and nuclear architecture patterns, which are associated with polygenic OR transcription during the INP stage (Fig. 4C). Dorsal OSNs only transcribe ORs with a dorsal identity during differentiation (Fig. 4C, top right), and these are the only ORs that are coated in heterochromatin and recruited to the repressive nuclear compartment in dorsal OSNs (Fig. 4B, bottom right). In contrast, ventral OSNs express ORs from all zones during the INP stage, and ORs from all zones are coated with heterochromatin and recruited to the repressive nuclear territory (Fig. 4C, left). Transcription during the polygenic OR stage may signal which class(es) of ORs are targets of heterochromatin and recruitment to the OR compartment. In ventral OSNs, OR transcription narrows to ventral ORs only in immature OSNs (Bashkirova et al., 2023). Lineage tracing supports this ‘wide to narrow’ zonal restriction pattern (Strotmann et al., 2009).

HiC is a chromatin conformation capture technique that measures DNA–DNA interactions across the genome and can be used to predict nuclear architecture. By performing HiC in zonally dissected tissue to measure OR to OR DNA contacts, Bashkirova and colleagues have uncovered a ‘sweet spot’ in the nucleus for OR expression (Bashkirova et al., 2023) (Fig. 4B). The number of inter-chromosomal contacts among OR clusters is associated with whether an OR can be expressed. High levels of OR–OR contacts indicate ORs buried in the repressive OR compartment. Low levels of OR–OR contacts indicate ORs that were never recruited to the OR compartment. In the anatomical zone where they can be expressed ORs exhibit an intermediate level of OR–OR contacts. ORs that are competent for transcription may be positioned at the edge of the repressive OR territory (sweet spot), which likely facilitates interaction with an OR enhancer hub.

#### Probabilistic OR choice

Within each zone, an OR allele from the zonal pool is chosen in a probabilistic manner. Symmetry breaking among candidate ORs appears to be governed by developmentally timed OR transcriptional upregulation. Computational work using single-cell RNA sequencing (scRNA-seq) demonstrates that high OR transcription during the immature OSN stage favors selection for monoallelic expression (Mohanty et al., 2023). Modulating OR promoter strength by introducing OR transgenes that add or remove TF binding sites changes the number of OSNs that express the transgenic OR (Rothman et al., 2005; Vassalli et al., 2011).

Early polygenic OR transcription is likely due to engagement with a *cis* OR enhancer (Pourmorady et al., 2024; Tan et al., 2019). *Trans* interactions increase among OR enhancers during the INP to immature OSN transition, resulting in several enhancer hubs per cell, of which only one becomes the active enhancer hub (Monahan et al., 2019; Pourmorady et al., 2024; Wu et al., 2024). The active hub has stronger HiC interactions with the expressed OR, is more euchromatic as determined by liquid chromatin HiC, and harbors H3K27ac marks (Belaghzal et al., 2021; Pourmorady et al., 2024; Wu et al., 2024). How a single active enhancer hub emerges from the multiple hubs per cell is currently unknown. The OR RNA could serve a non-coding role by recruiting or sequestering activating factors (Pourmorady et al., 2024). Alternatively, high levels of OR transcription could change local DNA secondary structures leading to non-canonical DNA structures (Makova and Weissensteiner, 2023), which protect the active OR from putative silencing factors. Curiously, the 3′UTR of the active OR allele is highly accessible, an unusual feature that might shed light on the OR–OR enhancer relationship (Monahan et al., 2017).

A model emerges from these data (Fig. 4B) whereby developmentally timed heterochromatin formation and nuclear architecture rearrangement allow for a subset of OR genes to come into the OR nuclear compartment. If an allele engages with a proximal OR enhancer, it may settle in the transcriptionally permissive OR territory at the edge of the repressive compartment. Should that OR successfully recruit *trans* enhancers and form an enhancer hub, OR transcription that is stochastically higher than other ORs in the cell could lead to formation of an active enhancer hub. Transcriptional upregulation of a single OR engaged with the active enhancer hub likely signals negative feedback, which is the defining state of immature OSN cells (Kahiapo, 2020).

### OR feedback

Monoallelic OR choice is stabilized by a negative feedback signal during the immature OSN stage that represses activation of other ORs in the same cell. A functional OR coding sequence is required to elicit feedback (Lewcock and Reed, 2004; Nguyen et al., 2007; Serizawa et al., 2003). The rapidly evolving OR gene family includes many pseudogenes (Niimura et al., 2014), selection of which would prevent an OSN from responding to odorants or projecting to stereotyped glomeruli. OSNs can switch OR expression from nonfunctional ORs to coding ORs, revealing the evolutionary elegance of feedback (Shykind et al., 2004).

The unfolded protein response (UPR) is the molecular mechanism underpinning the feedback signal in OSNs (Dalton et al., 2013). Only a functional OR protein can elicit UPR, and not a pseudogene. How the expression of a functional OR initiates UPR in OSNs is currently unknown, but might be a sudden increase in OR peptides, or a result of the absence of OR protein folding chaperones (Karagöz et al., 2019). UPR signaling leads to translation of the TF ATF5, which then enters the nucleus and activates downstream targets, including the OR protein chaperones RTP1 and RTP2 (Dalton et al., 2013; Kahiapo, 2020).

UPR leads to the expansion of the endomembrane system to ensure translation and trafficking of membrane-bound proteins, including ORs (Kaufman et al., 2002). Although UPR is generally thought of as a stress response pathway, it is activated normally during skin, immune and intestinal development (Coleman and Haller, 2019; Hetz and Papa, 2018; Sugiura et al., 2009). Once the cell has adapted its physiology, UPR is resolved, OSNs mature, and a single OR is translated at high levels and trafficked to the plasma membrane (Dalton et al., 2013; Gimelbrant et al., 2001; Saito et al., 2004). Therefore, UPR signaling serves a dual role to both ensure that only one OR is monoallelically expressed, and also to ensure that the OSN differentiates into a functional neuron.

Monogenic transcription of a functional OR is likely sufficient to signal negative feedback rapidly, preventing the activation of other ORs (Pourmorady et al., 2024). However, OR choice is plastic until feedback is resolved. That is, post-transcriptional mechanisms with known links to UPR can influence which OR is selected. Nonsense-mediated RNA decay can modulate OR choice, and may promote medial zonal OR expression (Tan and Xie, 2018; Tan et al., 2020). Without the OR protein chaperones RTP1 and RTP2, OR choice is skewed towards a smaller pool of 10% of ORs, highlighting that defects in OR trafficking can bias choice (Saito et al., 2004; Sharma et al., 2017). The mechanisms that enable OR switching during UPR merit further investigation. Could unresolved UPR delay OSN differentiation and lead to passive switching to a new OR-enhancer hub? Alternatively, could UPR signal back to enhancer hubs to derepress a dormant enhancer hub? It is known that ORs are expressed outside of their zones during the INP stage, but become spatially restricted during the immature OSN stage when UPR occurs (Bashkirova et al., 2023; Strotmann et al., 2009). Could feedback encode a signal that restricts OR expression to the appropriate zones?

UPR has the potential to delineate active and inactive OR enhancer hubs as they form in immature OSNs. OR enhancers have ATF5, CEBPG and BPTF motifs, inviting the hypothesis that these targets of UPR could bind and regulate OR enhancers (Kahiapo, 2020; Markenscoff-Papadimitriou et al., 2014). Future work will uncover the proteins that distinguish active from inactive enhancer hubs, and whether any of these factors are regulated by UPR feedback.

How does a functional OR protein signal that choice has been made, whereas a pseudogene cannot? The fact that OSNs can switch upon loss of OR protein chaperones (Sharma et al., 2017) suggests that the signal that ‘resolves’ UPR may come when an OR is properly folded or trafficked. Neuronal activity might also signal that a functional OR has been chosen (Dalton et al., 2013). A surprising finding reveals that immature OSNs depolarize in the presence of odorants (Huang et al., 2022), when UPR is active. Studies in which the DRY motif, necessary for OR signaling, has been mutated indicate that feedback can occur in the absence of neuronal activity (Imai et al., 2006; Nguyen et al., 2007). These transgenic experiments may not fully capture the role of neuronal activity if feedback is resolved by marking an enhancer hub, as it is unknown whether transgenic OR alleles interact with enhancer hubs. Further experiments should mutate DRY and other motifs with knock-in OR alleles that have *cis* regulatory regions intact.

### Maintenance of OR choice

Mature OSNs continue to translate the chosen OR for their lifetime, which ranges from weeks to one year (Holl, 2018; Kondo et al., 2010). Singular OR choice does not need to be maintained through mitosis, as the last cell division occurs before choice, during the INP stage (Fletcher et al., 2017). DNA methylation is not required for maintenance of OR choice (Colquitt et al., 2014). ORs are instead maintained in a repressed state by heterochromatin and recruitment to repressive nuclear foci (Clowney et al., 2012; Lyons et al., 2014). Importantly, mature OSNs lack the activating factors that promoted OR expression during the INP and immature OSN stages, including LSD1 (Lyons et al., 2013). This leads the repressed state to be stable; downregulation of a chosen OR in mature OSNs does not lead to derepression of other ORs (Abdus-Saboor et al., 2016).

How is the expression of the active OR allele maintained? Timely engagement with an active OR enhancer hub appears to be the most robust way to maintain monoallelic choice. Ectopically activating an OR by knocking-in a tetracycline transactivator (tTA) responsive tetO promoter into the endogenous OR locus leads the vast majority of OSNs to stably choose the knock-in allele, even if tTA is only transiently expressed during the INP/immature OSN stage (Bashkirova et al., 2023; Pourmorady et al., 2024). In striking contrast, without *cis*-regulatory elements that likely interact with an enhancer hub, the expression of a transgenic version of this construct is not maintained in mature OSNs (Nguyen et al., 2007). Ectopic OR expression can reverse choice to varying degrees, even in mature OSNs (Abdus-Saboor et al., 2016; Fleischmann et al., 2008; Nguyen et al., 2007; Pourmorady et al., 2024). Curiously, COVID-19 causes loss of OR expression and nuclear architecture rearrangement in OSNs, suggesting that infection can destabilize OR choice (Zazhytska et al., 2022). The signals that lead to the unraveling of stable OR choice either during infection or in experimental models are yet to be determined.

## Parallels and differences between OR and *Xist* regulation

Although the establishment of monoallelic expression occurs in very different contexts for *Xist* and OR genes, a series of parallels exists between the two systems. Clearly, symmetry breaking is a more elaborate process in OR choice, whereby one out of thousands of alleles must be chosen. OR choice is, therefore, a multistep process, in which only the last steps perform a stochastic choice among a limited number of OR alleles. However, both ORs and *Xist* transition through a ‘variable’ phase, during which cells might co-express multiple alleles transiently. This might indicate that a very rare and infrequent symmetry-breaking event driving stochastic monoallelic upregulation in all cells is either difficult to evolve or would make the process too slow. Instead, different regulatory layers provide a series of symmetry-breaking opportunities, and corrective mechanisms are in place when symmetry breaking fails or a non-functional allele is chosen. To trigger negative feedback, cells sense the physiological consequence of the monoallelic genes: X-linked gene silencing and OR protein expression, respectively. Moreover, successful monoallelic expression promotes further cell differentiation in both systems, suggesting a tight coupling between cell state and the monoallelic process. Once the monoallelic state is established, it must be maintained in both cases, but over very different timescales. OR expression must only be maintained on the scale of several months to a year, and occurs in the absence of cell division, because the tissue is constantly regenerating. Monoallelic expression of *Xist*, in contrast, is maintained through many cell cycles up to decades. This might explain why DNA methylation, which can be inherited through mitosis, plays a role in *Xist* regulation, but is absent from OR genes. Interestingly, both systems use the repressive histone modification H3K9me3 to keep the non-selected alleles silent. The molecular events that truly carry epigenetic memory remain to be dissected. ORs and *Xist* are thus fascinating examples of random, but stable, monoallelic expression, and the governing mechanisms that have been discovered over the years may shed light on less well-studied incidences of monoallelic expression.

As the field moves forward, it will be useful to explore how the mechanisms directing monoallelic choice in XCI and OSN differentiation apply to other monoallelic expression systems, including imprinted genes, protocadherin promoter choice and antigen receptor choice in immune cells. Several of the characteristics of *Xist* have been identified as general features of human RME genes, such as antisense transcription, intragenic regulatory elements and multiple enhancers (Kravitz et al., 2023). In addition, non-coding RNAs play essential roles at imprinted loci (Zwart et al., 2001), and have recently been shown to direct protocadherin promoter choice (Canzio et al., 2019). Nuclear architecture plays instructive roles in monoallelic systems (Canzio et al., 2019; Loftus et al., 2023; Proudhon et al., 2016), highlighting potentially common upstream signals among the monoallelic paradigms that orchestrate changes in nuclear architecture. As protocadherins and antigen receptors are both expressed at the cell surface in differentiating cell types, similar to ORs, it would be interesting to investigate whether UPR, with its known participation in cellular differentiation and membrane-bound protein expression, plays a role in feedback, locking in monoallelic choice in other systems.
